# Frailty and associated factors among older patients with non-dialysis chronic kidney disease: a multicenter cross-sectional study in three hospitals in Yaounde, Cameroon

**DOI:** 10.11604/pamj.2025.52.116.48268

**Published:** 2025-11-18

**Authors:** Marie-Josiane Ntsama Essomba, Serge Patrick Medoua Bella, Nzana Victorine Bandolo, Maimouna Mahamat, Aristide Nono, Emmanuelle Ndjong, Hermine Fouda, Gloria Ashuntantang, François Kaze Folefack

**Affiliations:** 1 Department of Internal Medicine and Specialties, Faculty of Medicine and Biomedical Sciences, University of Yaounde I, Yaounde, Cameroon,; 2 Nephrology Unit, Yaounde University Teaching Hospital, Yaounde, Cameroon,; 3 Nephrology Unit, Yaounde General Hospital, Yaounde, Cameroon,; 4 Department of Internal Medicine and Specialties, Faculty of Health Sciences, University of Bamenda, Bambili, Cameroon

**Keywords:** Frailty, chronic kidney disease, geriatric epidemiology

## Abstract

**Introduction:**

frailty is common in older patients with chronic kidney disease (CKD) and is associated with poor quality of life and adverse clinical outcomes. This study aimed to determine the prevalence of frailty among older patients with non-dialysis CKD (ND-CKD) and to determine associated factors in Cameroon.

**Methods:**

this cross-sectional study was conducted in three hospitals in Cameroon, including patients with ND-CKD aged ≥60 years. The 2009 CKD Epidemiology Collaboration (CKD-EPI) equation was used to estimate the glomerular filtration rate (eGFR). Frailty was assessed using Fried´s criteria. A multivariate regression analysis was performed, and a p-value ≤0.05 was statistically significant.

**Results:**

we included 102 participants (60.8% male) with a median age (interquartile range (IQR)) of 69 (65-73) years. The prevalence of frailty was 35.3%. Compared to patients without frailty, frail patients were older, had lower education, had lower incomes, had a lower eGFR and a lower body mass index (BMI), had a previous stroke, a previous fracture, activities of daily living (ADLs) dependency, had lower muscle strength, and a slower gait speed. Patients with previous stroke (adjusted odds ratio (aOR) 3.9, 95%CI (95% confidence interval) 1.1-13.9) and ADL dependency (aOR 28.2, 95%CI 3.3-235.2) were likely to be frail. Frailty was less frequent in patients with an income-producing activity (aOR 0.05, 95%CI 0.01-0.67).

**Conclusion:**

frailty was common in this group of older patients with ND-CKD from Cameroon. Further actions are needed to increase the screening of patients at risk of frailty to tailor interventions, taking into account available resources.

## Introduction

Older people are at high risk of developing chronic kidney disease (CKD) owing to physiologic age-related decline in kidney function, combined with traditional risk factors including hypertension, diabetes, and obesity [1]. The burden of CKD in Africa is underestimated mainly due to the scarcity of reliable data. The prevalence in the general population varies between 4 to 16% and is higher in sub-Saharan Africa than in North Africa [2]. In 2021, the prevalence of CKD amongst older people was 40% compared to 13.4% in the general population worldwide [3,4]. In Uganda, the prevalence of CKD was 11% amongst people aged ≥60 years, with an increase of 3.5-fold risk increase by older age [5]. In Cameroon, the prevalence of CKD varies between 3 [6] and 14.1% [7], with advanced age as an associated factor [8].

Frailty has emerged as a common syndrome among older adults and is associated with an increased risk of poor health outcomes, including functional decline, falls, institutionalization, and death [9,10]. Although it can be assessed by various methods, the Fried Phenotype Model of frailty, which focuses on physical frailty, is the most common method of frailty assessment in CKD [11-13]. Patients with CKD are more likely to develop physical frailty. Although their relationship is not completely understood, CKD and frailty share common metabolic changes, including accumulation of advanced glycation end products, insulin resistance, oxidative stress, and chronic inflammation [14-17]. Furthermore, reduced energy intake, which is frequent in CKD patients, can contribute to sarcopenia and, subsequently, to physical frailty [18,19]. Decreased 1,25-dihydroxyvitamin D associated with CKD also contributes to frailty in this population [20].

According to a systematic review conducted in 2023, about 35% of patients with CKD showed signs of frailty [21]. In older patients with non-dialysis CKD (ND-CKD) sub-populations, the prevalence of frailty varies from 9 to 11.9% in stage 3 and stages 4-5 CKD [22]. Frailty is associated with adverse clinical outcomes and an increased risk of mortality and hospitalization in all stages of CKD [21,23]. Despite much evidence suggesting that frailty is associated with poor outcomes, frailty screening has not been widely incorporated in routine nephrology care in our setting. Furthermore, little is known about frailty among older people with CKD in Africa. Hence, this study aimed to determine the prevalence and associated factors of frailty among older patients with ND-CKD.

## Methods

**Study design:** we conducted a cross-sectional study to determine factors associated with frailty in ND-CKD older patients.

**Study setting and population:** patients were recruited from June to September 2022 in three referral hospitals of the City Capital of Cameroon, the Yaoundé General Hospital, the Yaoundé University Teaching Hospital, and Yaoundé Central Hospital. These hospitals have been chosen as they are the only ones to have an outpatient nephrology consultation. We included patients aged ≥60 years with ND-CKD stage 3 to 5 who provided an informed written consent. We excluded patients who required dialysis and those presenting with clinical conditions that would impede physical assessment, such as severe dementia, Parkinson´s disease, cancer, or severe disability.

**Variables:** variables included age, sex, marital status, and employment status. Also, variables included body mass index, comorbidities, medications used, functional status, history of CKD, etiologies of CKD, serum creatinine, estimated glomerular filtration rate, and hemoglobin level. The outcome variable was frailty. Independent variables included age, sex, comorbidities, etiologies of CKD, estimated glomerular filtration rate, and functional status.

### Data resource and measurement

**Data collection:** a questionnaire was used to collect sociodemographic (age, sex, marital and employment status), clinical (body mass index (BMI), comorbidities, medications used, history of CKD, etiologies of CKD), and paraclinical (serum creatinine, hemoglobin level) data. We recorded the last value of haemoglobin level reported in the file (less than one week before inclusion). Anemia was defined as a hemoglobin level ≤10g/dl. Polymedication was defined as the concomitant use of more than 5 medications.

**Evaluation of kidney function:** the diagnosis of CKD was made by the treating nephrologists using the last value of serum creatinine (less than 1 week before inclusion) in the records of the patients. The 2009 CKD Epidemiology Collaboration (CKD-EPI) equation was used to estimate the glomerular filtration rate and classified based on the Kidney Disease Improving Global Outcomes (KDIGO) criteria as followed: as stage 3 for an estimated glomerular filtration rate (eGFR) of 30-59 mL/min/1.73 m^2^, stage 4 for an eGFR of 15-29 mL/min/1.73 m^2^, and stage 5 for an eGFR < 15mL/min/1.73 m^2^ [24].

**Assessment of frailty:** frailty was defined based on Fried´s criteria, as the presence of at least 3 of 5 of the following conditions: unintentional weight loss, weakness, slow walking speed, low physical activity, and exhaustion [11,25]. Weight loss was defined as an unintentional loss of ≥5kg during the 12 past months. Muscle strength was assessed by the grip strength measured in the patient´s dominant hand using an electronic Jamar Plus® dynamometer. We reported the average value from 3 consecutive attempts. Weakness was defined as a grip strength <20kg for women and <30kg for men. Walking speed was assessed by measuring the 4-m gait speed at the normal pace of each participant. We reported the average of 2 attempts for each participant. Gait speed <1m/s was considered a slow walking speed. Physical activity was assessed by self-reported exercise habits. Participants who reported «never» or «rarely» being active at least once a week were categorized as physically inactive. Exhaustion was defined by self-report generalized weakness or lack of energy.

**Assessment of functional status:** functional status assessment was derived from the Katz *et al*. activities of daily living (ADLs) tool [26]. Dependency was defined as the presence of one or more incapacity to perform ADLs.

**Sample size:** sample size was calculated using the OpenEpi sample size calculator available on the OPENEPI website, using the prevalence of frailty among ND-CKD patients, giving a minimal sample size of 101 participants [22].

**Data analysis:** data were analyzed with the Statistical Package for Social Sciences for Windows (SPSS 23.0, Chicago, Illinois, USA). Quantitative variables are presented as mean and standard deviation (SD) or median and interquartile range (IQR). Categorical variables are presented as frequencies and proportions. Means or medians were compared using the Student´s T test or U-Mann-Whitney test when appropriate, and proportions using the Chi-squared test or Fisher's exact T test. Variables that showed P <0.1 in the univariable analysis were included in the multivariable logistic regression analysis, which estimated the odds ratio (OR) and 95% confidence interval (CI) with the non-frail group as the reference group. A P <0.05 was used to define statistical significance.

**Ethical considerations:** ethical approval was obtained from the Institutional Review Board of the Faculty of Medicine and Biomedical Sciences of the University of Yaounde I, under the reference number N°365/UY1/FMSB/VDRC/CSD. All the patients have given their consent to participate.

## Results

**Sociodemographic analysis:** we included 102 ND-CKD participants, of whom 60.8% were males. The median (IQR) age was 69 (65-73) years, with an age range from 60 to 86 years. Other sociodemographic characteristics are presented in Table 1.

**Table 1:** sociodemographic factors associated with frailty among older patients with non-dialysis-chronic kidney disease recruited from the nephrology units of Yaounde from June to September 2022 (N=102)

	Frail n=36(%)	Non-frail n=66(%)	All n=102(%)	uOR (95%CI)	P	aOR (95%CI)	P
Male^a^	14 (38.9)	26 (39.4)	40 (39.2)		1.000		
Female	22 (61.1)	40 (60.6)	62 (60.8)				
Median age (IQR)^b^	72 (66-78)	68 (65-72)	69 (65-73)	-	0.07	-	0.127
**Marital status^a^**							
Single	7 (19.4)	15 (22.7)	22 (21.6)	-	0.804	-	-
In relationship	29 (80.6)	51 (77.3)	80 (78.4)	-			
**Educational level^a^**							
None	7 (19.4)	3 (4.5)	10 (9.8)	5.1 (1.22-21.01)	0.031	-	0.294
Primary	15 (11.7)	18 (27.3)	33 (32.4)	-	0.184	-	-
Secondary	11 (30.6)	32 (48.5)	43 (42.2)	-	0.096	-	-
**Income-producing activity^a^**							
Yes	1 (2.8)	16 (24.2)	17 (16.7)	0.1 (0.01-0.71)	0.011	0.05 (0.01-0.67)	0.023
No	35 (97.2)	50 (75.8)	85 (83.3)				

ADL: activities of daily living; aOR: adjusted odds ratio; CVD: cardiovascular disease; Hx: history, 95%CI: 95% confidence interval; IQR: intequartile range; SD: standard deviation; uOR: unadjusted odds ratio; ^a^categorical variables are shown as number of cases (percentage); ^b^continuous variables are shown as median and interquartile range; ^c^continuous variables are shown as mean and standard deviation; all variables with a p-value at the 0.1 threshold have been included in the final model; the p-values indicated in bold are statistical significant

**Descriptive analysis:** the majority of participants were overweight (49%), and the mean BMI was 27.6 ± 5.1 kg/m^2^. Polymedication was present in 62.7% of participants, whereas 11.8% were dependent on ADL. Anemia was present in 29.4% of participants. Other clinical characteristics are presented in Table 2. Concerning the history of the CKD, the median (IQR) duration of follow-up since the diagnosis was 17.5 (4-37) months. The mean eGFR (SD) of the study population was 25.7ml/min/1.73m^2^ (11.9). Participants were classified as CKD stage 3 (40.2%), stage 4 (37.3%), and stage 5 (22.5%). The main etiologies of CKD were hypertension (27.5%) and ischemic nephropathy (24.5%) (Table 3). The prevalence of frailty was 35.3%. The main components of frailty were slowness (89.2%%), physical inactivity (81.4%), and exhaustion (68.6%). The prevalence of frailty stratified by stage of CKD was 30.6% in stage 3 and 69.4% in stage 4 and 5.

**Table 2:** clinical factors associated with frailty among older patients with non-dialysis-chronic kidney disease recruited from the nephrology units of Yaounde from June to September 2022 (N=102)

	Frail n=36 (%)	Non-frail n= 66 (%)	All N=102(%)	uOR (95%CI)	P	aOR (95% CI)	P
Mean BMI in kg/m^2^ (SD)^c^	25.9 (6.1)	28.6 (4.2)	27.6 (5.1)	-	0.049	-	0.064
**BMI (kg/m^2^) classification^a^**							
<18.5	4 (100)	0 (0)	4 (3.9)	0.33 (0.25-0.43)	0.014	-	0.999
18.5 - 24.9	11 (30.6)	13 (19.7)	24 (23.5)	-	0.271	-	-
25 - 29.9	16 (44.4)	34 (51.5)	50 (49)	-	0.495	-	-
≥ 30kg	5 (13.9)	19 (28.8)	24 (23.5)	-	0.142	-	-
**Comorbidities^a^**							
Hypertension	32 (88.9)	64 (97)	96 (94)	-	0.181	-	-
Osteoarthritis	27 (75)	42 (63.3)	69 (67.7)	-	0.275	-	-
Diabetes	20 (55.6)	34 (51.5)	54 (52.9)	-	0.836	-	-
CVD	3 (8.3)	10 (15.2)	13 (12.7)	-	0.373	-	-
Hx of previous stroke^a^	11 (30.6)	7 (10.6)	18 (17.7)	3.71 (1.29-10.8)	0.016	3.9 (1.1-13.9)	0.036
Hx of previous Fracture^a^	4 (11.1)	1 (1.5)	5 (4.9)	8.13 (1.00-75.7)	0.05	-	0.235
**Polypharmacy^a^**							
Yes	25 (69.4)	39 (59.1)	64 (62.8)	-	0.392	-	-
No	11 (30.6)	27 (40.9)	38 (37.3)				
**ADL dependency^a^**							
Yes	11 (30.6)	1 (1.5)	12 (11.8)	28.6 (3.51-233.2)	< 0.001	28.2 (3.3-235.2)	0.002
No	25 (69.4)	65 (98.5)	90 (88.2)				
Anemia (yes)	14 (38.9)	16 (24.2)	30 (29.4)	-	0.172	-	-

ADL: activities of daily living; aOR: adjusted odds ratio; CVD: cardiovascular disease; Hx: history, 95%CI: 95% confidence interval; IQR: intequartile range; SD: standard deviation; uOR: unadjusted odds ratio; ^a^categorical variables are shown as number of cases (percentage); ^b^continuous variables are shown as median and interquartile range; ^c^continuous variables are shown as mean and standard deviation; all variables with a p-value at the 0.1 threshold have been included in the final model; the p-values indicated in bold are statistical significant

**Table 3:** characteristics of chronic kidney disease and association with frailty in older patients with non-dialysis-chronic kidney disease recruited from the nephrology units of Yaounde from June to September 2022 (N=102)

	Frail n=36 (%)	Non-frail n= 66 (%)	All n=102 (%)	uOR	95% CI	P	aOR	95% CI	P
Mean eGFR ml/min /1.73m^2^(SD)^a^	22.5 (8.1)	27.4 (13.4)	25.7 (11.9)	-	-	< 0.001	-	-	0.156
**Etiologies of CKD^a^**									
Chronic glomerulonephritis	2 (5.6)	3 (4.5)	5 (4.9)	-	-	1.000	-	-	-
Hypertension	9 (25)	19 (28.8)	28 (27.5)	-	-	0.817	-	-	-
Diabetic nephropathy	3 (8.3)	3 (4.5)	6 (5.9)	-	-	0.663	-	-	-
Ischemic nephropathy	8 (22.2)	17 (25.8)	25 (24.5)			0.811	-	-	-
Chronic tubulointerstitial nephritis	8 (22.2)	11 (16.7)	19 (18.6)	-	-	1.000	-	-	-
**CKD stage^a^**									
3	11 (30.6)	30 (45.5)	41 (40.2)	-	-	0.205	-	-	-
4	17 (47.2)	21 (31.8)	38 (37.3)	-	-	0.093	-	-	-
5	8 (22.2)	15 (22.7)	23 (22.5)	-	-	0.580	-	-	-

aOR: adjusted odds ratio; CKD: chronic kidney disease; eGFR: estimated glomerular filtration rate; SD: standard deviation; uOR: unadjusted odds ratio; ^a^: categorical variables are shown as number of cases (percentage)

**Bivariate analysis:** compared to patients without frailty, frail patients were older, had lower education, had lower incomes, had a lower eGFR and a lower BMI, had a previous stroke, a previous fracture, ADL dependency, had a lower muscle strength, and a slower gait speed.

**Multivariate analysis:** in multivariable logistic regression analysis, frailty was more commonly associated with a history of stroke (adjusted odds ratio (aOR) 3.9 95%CI (95% confidence interval) 1.1-13.9) and ADLs dependency (aOR 28.2 95%CI 3.3-235.2), whereas it was less frequent in patients with an income-producing activity (aOR 0.05 95%CI 0.01-0.67).

## Discussion

Our study is the first to report frailty among patients with ND-CKD in Cameroon. We aimed to determine the prevalence of frailty and associated factors among older patients with ND-CKD. In our study, the prevalence of frailty was 35.3%. The most common components of frailty were slowness, physical inactivity, and exhaustion. The frail group and the non-frail group differed in terms of age, mean eGFR, educational level, income-producing activities, BMI, previous stroke, previous fracture during the past 12 months, and ADL dependency. Frailty was more commonly associated with a history of stroke and ADL dependency, whereas it was less frequent in patients with an income-producing activity. The prevalence of frailty in our study was similar to the prevalence reported in Brazil and Korea [27,28]. However, this is higher than the 7% reported by Reese *et al*. in a study that included community-dwelling older patients with CKD, with a median age of 65 years [29]. Our prevalence is also far from the 14% reported among pre-dialysis outpatients in the USA, with a median age of 59 years [23]. In another study conducted by Rodriguez *et al*. no patient was found to be frail [30]. This discrepancy suggests that the identification of frailty depends on the method of assessment but also on the characteristics of the population. Indeed, previous studies on frailty in older patients with CKD have been conducted among community-dwellers, where the prevalence and severity of CKD are low [29,31]. Suggesting also the impact of the metabolic disturbances associated with CKD that can impede with physical performances.

We found a difference between frail and non-frail older patients with ND-CKD with respect to age and eGFR. Indeed, the frail group was older and had a lower mean eGFR. It has been consistently reported that the prevalence of frailty increases with age [11] and decreased eGFR [23,28,32]. Wilhelm *et al*. found that the odds of frailty for persons with stage 5 CKD were 6.7 compared to 2.6 in stage 3b [32]. The frail patients in our study were older than those in a study involving stage 5 CKD patients (69 vs 58.2 years old) [33]. This finding suggests that, regarding the influence of age and eGFR, people with more severe CKD are more likely to be frail.

The main components of frailty in our study were physical inactivity, slowness, and exhaustion. These findings are in line with previous studies [23,29]. This suggests evidence for muscle dysfunction and low physical performance in our study population, demonstrated by a high prevalence of slow walking speed but also by the presence of low muscle strength in all stages of CKD. Low physical performance and low muscle strength are both hallmarks of sarcopenia, a condition characterized by an accelerated loss of muscle mass and function, commonly, but not exclusively, associated with age [34,35]. CKD is both a cause and an accelerating factor of sarcopenia. Indeed, muscle structure and metabolic function are adversely affected by chronic inflammation, protein-energy wasting, and insulin resistance in patients with CKD [15,18,36]; all of which overlap with phenotypic impairments associated with frailty [37,38].

Both frail and non-frail participants were found to be overweight and physically inactive. However, the frail group was likely to have a lower BMI than the non-frail participants. This is consistent with other findings [23,27], although Yang *et al*. reported different findings with higher BMI being associated with a higher likelihood of being frail [22]. High BMI is frequent among CKD patients in Cameroon [7,39]. However, this finding must be interpreted with caution, as CKD patients´ weight is also affected by fluid retention. Frailty is described as a wasting disorder with diagnosis criteria including weight loss and low muscle strength [17,38], but several studies have reported high prevalence of frailty in overweight and obese persons [40,41]. Obesity can independently lead to loss of muscle mass and function, due to the negative impact of adipose tissue-dependent metabolic disturbances, such as oxidative stress, inflammation, and insulin resistance [42]. Furthermore, lifestyle interventions with weight loss targeting excess fat can also lead to loss of skeletal muscle mass, which may be more pronounced in individuals with predisposing catabolic conditions like CKD. Physical inactivity may also play a relevant role, being a cause and a consequence of both skeletal muscles wasting and overweight.

We found no difference with respect to frailty prevalence between men and women, which is consistent with other findings [22,28]. However, we found that frail and non-frail groups differed in terms of educational level and income-producing activities. This is different from findings in Korea, where social characteristics, including educational level and living alone, were not associated with frailty [28]. However, our findings are in line with a study conducted in Sweden, where older people with the lowest education level accounted for the greatest proportion of frail individuals [43]. In the Netherlands, low educational level was associated with the highest odds of being frail in comparison to higher education level [44]. This is also supported by a meta-analysis conducted in 2021 [45]. Limited formal education is still common in the older population in Cameroon. The impact of socioeconomic deprivation in adults with ND-CKD is complex. There is evidence that patients with CKD who are socioeconomically deprived have faster disease progression, higher risk of cardiovascular disease and premature mortality [46]. Poor socioeconomic status is also associated with higher frailty prevalence. Indeed, frailty occurs earlier and progresses more rapidly with lower socioeconomic position [47]. In our study, having an income-producing activity could suggest more financial resources to afford healthcare expenditures, but also adequate nutrition. This demonstrates that improvement of socioeconomic status could be a key to preventing or delaying frailty in older patients with ND-CKD.

We found that a history of stroke was independently associated with an increased risk of frailty among older patients with ND-CKD. In a meta-analysis conducted in 2022, Burton *et al*. showed that frailty is common in patients presenting with acute stroke [48]. In a prospective cohort study conducted in 2019, Tollitt *et al*. has shown that patients with CKD who suffered a previous stroke had a higher risk of adverse clinical outcomes than those without stroke during their follow-up [49]. The neurological impairments following stroke are likely to exacerbate the phenotypic characteristics of frailty, especially gait speed, muscle strength, and weight loss [50]. Despite improvement in stroke care in Cameroon, stroke can leave survivors with disabilities, requiring permanent assistance for ADLs. Thus stroke can accelerate the transition from robustness to frailty due to poor recovery.

Our study had several limitations, the first being the study design. Indeed, the cross-sectional design did not allow us to determine the relationship between frailty and dialysis initiation since there was no follow-up. Furthermore, we did not perform blood analysis; instead, we used the serum creatinine values already available in the patients' records. Second, despite the fact that we included patients from three different sites, our sample size is not large enough to generalize our findings.

## Conclusion

In this study, frailty was common, affecting more than one-third of participants. It was significantly associated with functional dependency in ADLs and a history of stroke, while it was less frequent among participants engaged in income-producing activities. These findings highlight the link between frailty and reduced physical as well as social functioning in this population. Identifying frailty and its correlates within ND-CKD patients is essential for optimizing clinical management and guiding supportive interventions aimed at preserving functional independence and improving socioeconomic support.

### What is known about this topic

- Frailty is common among older patients with CKD;
- Individuals who have CKD often present with signs and symptoms that may be consistent with the frailty phenotype.

### What this study adds

- To the best of our knowledge, this is the first study to report frailty assessment among older patients with ND-CKD in Cameroon;
- Our findings suggest that clinical conditions that impair the phenotypic characteristics of frailty, like previous stroke, but also poor socioeconomic status and educational level, can worsen the trajectory of ND-CKD older patients from robustness to frailty in Cameroon.
